# Shifting waters: how habitat alteration affects host-parasite dynamics in freshwater ecosystems

**DOI:** 10.1007/s00436-026-08730-1

**Published:** 2026-07-15

**Authors:** Bernd Sures, Annabell Hüsken, Sebastian Prati, Jessica Schwelm, Daniel Grabner

**Affiliations:** 1 Aquatic Ecology, University of Duisburg-Essen, Essen, Germany; 2 Centre for Water and Environmental Research, University of Duisburg-Essen, Essen, Germany; 3 Research Center One Health Ruhr, Research Alliance Ruhr, University of Duisburg- Essen, Essen, Germany; 4 Water Research Group, Unit for Environmental Sciences and Management, North- West University, Potchefstroom, South Africa; 5 UMR Entropie, Université de La Réunion, Saint-Denis, Réunion Island France

**Keywords:** Parasite ecology, Global change, Parasite decline, Zoonosis, Degradation

## Abstract

Human activities have profoundly altered freshwater ecosystems, reshaping host-parasite dynamics with sometimes far-reaching ecological and public health consequences. Based on the examples of stream degradation and restoration, dam construction, agriculture, and urbanization, this review synthesizes how habitat alteration influences parasite communities in freshwater systems. Restoration efforts, such as those in Germany’s Emscher River, demonstrate partial recovery of parasite diversity, though persistent stressors often favour generalist taxa with simple life cycles, reflecting incomplete ecosystem recovery. Dams and reservoirs, by contrast, frequently amplify parasite transmission by creating lentic habitats that aggregate hosts and vectors, increasing risks for diseases such as schistosomiasis and malaria. Agricultural land use further complicates these dynamics: while eutrophication and irrigation can boost trematode transmission, agrochemicals and habitat fragmentation may depauperate parasite communities or disrupt complex life cycles. Additionally, urbanization-related stressors, such as pollutants and artificial light, can suppress sensitive parasites while benefiting those adapted to disturbed environments. Our review demonstrates that, under habitat alteration, freshwater parasites fulfil multiple roles: they constitute a component of the impacted biodiversity, serve as indicators of ecosystem health, and influence disease risk, with their responses governed by life cycle complexity, host specificity, and environmental resilience. These findings underscore the need for integrated management strategies that account for parasites in conservation and public health planning.

## Introduction

Anthropogenic activities have massively shaped and changed the face of the Earth. Today, between 75 and 95% of the global terrestrial ice-free surface is estimated to be impacted by human activities, affecting both terrestrial and aquatic ecosystems and biodiversity (Ellis 2021). These impacts affect not only free-living species but also the diversity and abundance of parasites (Cable et al. 2017; Sures et al. 2023). Changes in land use can lead to a decline in parasite diversity or an increase in parasite abundance, which might result in detrimental health effects for humans and livestock (Cable et al. 2017; Gottdenker et al. 2014; Wood 2024). Besides their role as disease agents, parasites are important for the functioning of ecosystems (Louvard et al. 2025; Marcogliese 2004, 2005; Sures et al. 2017a), and therefore the significant decline in parasite diversity of the last decades is alarming (Gomez and Nichols 2013; Sures et al. 2023; Wood et al. 2023).

**Fig. 1:**
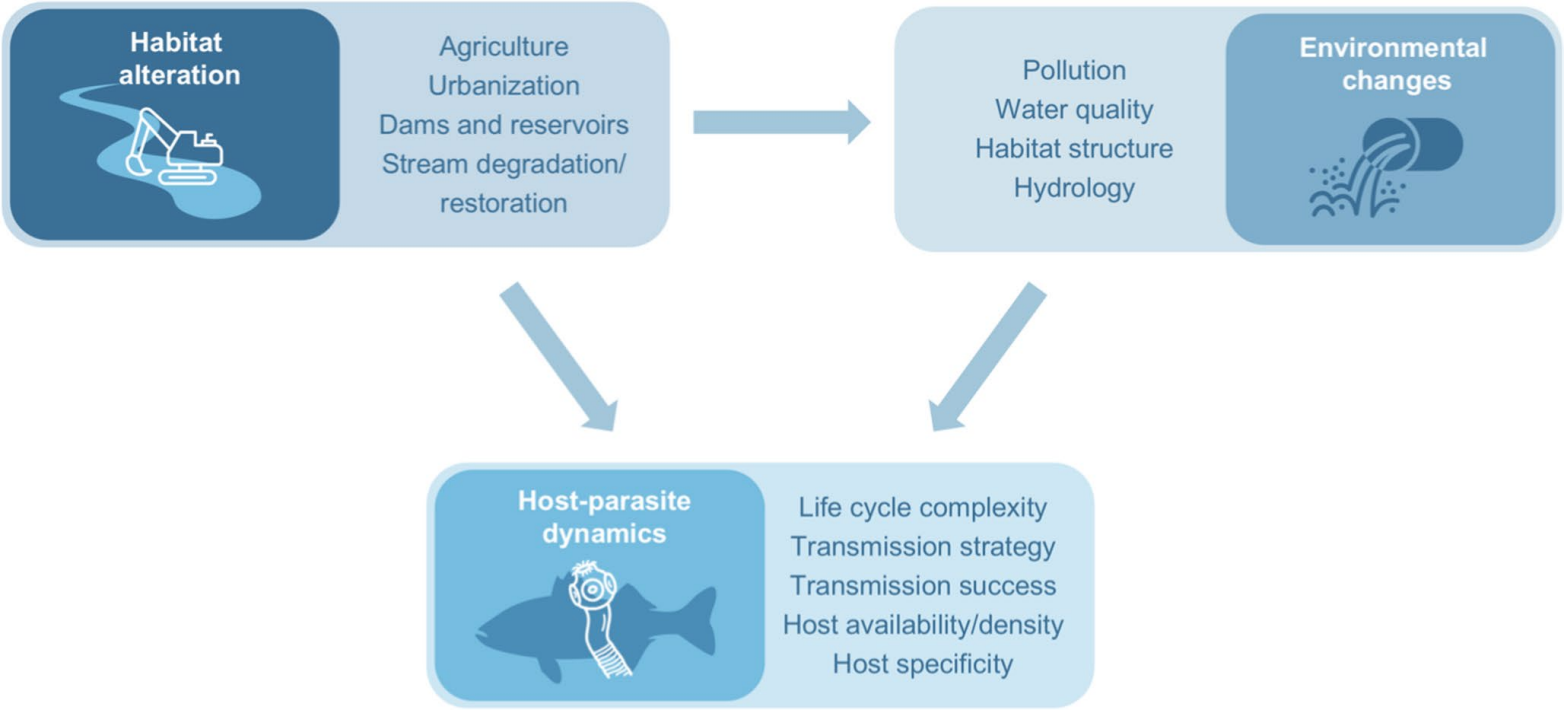
Illustration of the major effects of habitat alterations on parasite transmission

To illustrate the impact of habitat alteration on parasites, we examined selected examples of human-induced habitat change and their consequences for the development of parasite communities, focusing on freshwater ecosystems. We addressed stream degradation and restoration, dams and reservoirs as well as agriculture and urbanization as major causes of habitat alteration and highlighted the related ecological and health effects of parasites (Fig. 1).

## Stream degradation and restoration

Faced with the rampant degradation of watercourses and the resulting biodiversity loss, wastewater management measures have been implemented, and numerous local restoration initiatives have sprouted across Europe. These efforts have led to significant improvements in water quality and habitat structure and, consequently, to a partial recovery of benthic invertebrate assemblages (Haase et al. 2023; Schürings et al. 2025). However, progress in this sense has slowed in recent years, likely due to the limited extent of hydromorphological restoration and the ongoing presence of external stressors, e.g., related to land use, that hinder recovery progress (Haase et al. 2023).

One such example for freshwater degradation and restoration is the Emscher River in western Germany, once considered among the most polluted and hydromorphologically degraded river systems in Europe (Seidel et al. 2022). For decades, many of its tributaries as well as the main stem were used as concrete open sewage canals, leaving them almost devoid of life, except for highly tolerant taxa such as oligochaetes and bacteria (Winking et al. 2014). Following its gradual restoration between 1993 and 2023, biodiversity, including parasitic species, showed clear signs of recovery (Gillmann et al. 2023; Hüsken et al. 2026; Prati et al. 2022; Winking et al. 2014). Both host and parasite communities in restored reaches have diversified, becoming increasingly similar to those found in more pristine ecosystems. However, the persistent presence of anthropogenic stressors likely impedes complete ecological recovery. These dynamics are reflected in the composition of parasite communities within the system, differing in life cycle complexity (i.e., the number of hosts involved in the life cycle), transmission strategies, and the degree of host specialization, consistent with the Asymmetric Recovery Concept (ARC) proposed by Vos et al. (2023). Briefly, the ARC theorizes that parasites with simple life cycles and host generalists dominate in early stages of ecosystem recovery, whereas parasites with more complex life cycles and higher host specificity increase in diversity and dominance as recovery progresses (Sures et al. 2023). In the Boye catchment, a restored tributary of the Emscher, these processes can be exemplified by microsporidian and trematode parasites, which typically exhibit simple and complex life cycles, respectively. Diversity and abundance of directly transmitted microsporidians in amphipod hosts in the system were high and consisted exclusively of host generalists, indicating that, despite substantial restoration efforts, ecosystem recovery has not yet reached a climax (Prati et al. 2022). Likewise, trematode communities in the first intermediate snail hosts had high prevalence but were restricted to taxa transmitted by mobile definitive hosts such as waterfowl (Hüsken et al. 2026). Accordingly, all trematode taxa in the catchment were associated with allogenic life cycles characterized by high dispersal capacity, while no autogenic taxa, i.e., parasites completing their life cycle entirely within the aquatic habitat, were detected, potentially indicating incomplete recovery of local food webs (Esch et al. 1988; Marcogliese 2005).

## Effects of dams and reservoirs on parasite transmission

Dams and reservoirs are among the most omnipresent anthropogenic alterations of freshwater ecosystems and can profoundly reshape host-parasite dynamics. By transforming lotic systems into lentic or semi-lentic environments, dam construction modifies hydrology, habitat structure, and biotic communities, with consequences that may either enhance or suppress parasite transmission. Empirical evidence indicates that reservoirs can either function as hotspots of parasite exposure, increasing the prevalence of certain parasitic diseases, including neglected tropical diseases (NTDs) (Le et al. 2025; Ong et al. 2016; Sokolow et al. 2017), or lead to reduced transmission following large-scale environmental change (Kyei-Baafour et al. 2020; Zhang et al. 2019).

A primary mechanism through which dams and reservoirs affect parasite transmission is host aggregation. Artificial water bodies act as focal points for wildlife, livestock, and human activities, particularly in landscapes with limited access to water. Such aggregation increases contact rates between hosts and environmentally transmitted parasite stages, thereby amplifying exposure risks through drinking, grazing, bathing, or occupational contact even when host density at the landscape scale remains unchanged (Titcomb et al. 2021). Another mechanism by which dam construction can affect parasites is the shift in vector populations (e.g., mosquitoes, snails) due to alterations in local ecosystems (Patz et al. 2000).

Trematodes may be among the parasite groups most strongly affected by dam-induced habitat alterations, largely due to their reliance on freshwater snails as first intermediate hosts. The formation of slow-flowing or stagnant waters within reservoirs and adjacent areas provides favourable conditions for the proliferation of medically and ecologically important snails, which benefit from reduced flow velocity, increased macrophyte cover, and stabilized shorelines (Diakité et al. 2017; Sokolow et al. 2017). The often eutrophic conditions in mature reservoir systems, such as those in Germany’s Ruhr River, further promote trematode diversity (Selbach et al. 2020; Soldánová et al. 2010). Multiple studies document elevated trematode infection rates in reservoir systems, although patterns are not universally consistent. Investigations of hydropower reservoirs in Vietnam revealed high trematode prevalence (nine cercarial morphotypes) in thiarid and bithyniid snails, including *Melanoides tuberculata*, *Gabbia fuchsiana*, and *Parafossarulus manchouricus*, with infection rates ranging from 2.9% to 15.4% (Le et al. 2025). Similarly, prevalence and intensity of infection in fish in reservoirs in Thailand’s Ubolrantana River were notably higher than in lotic fishes in the area, due to changes in fish species assemblages (Ong et al. 2016). Additionally, reservoir environments can increase contact rates between free-swimming cercariae and fish or crustacean second intermediate hosts, as observed in Mekong River reservoirs where aquaculture practices amplified transmission of the small liver fluke *Opisthorchis viverrini* by concentrating infected fish populations. As a consequence, the rate of human infections with *O. viverrini* increased in the area (Ong et al. 2016; Ziegler et al. 2013). Dam construction also increases the risk of schistosomiasis in humans by excluding snail predators, such as river prawns, leading to explosive increases in snail populations and, consequently, schistosomiasis transmission (Sokolow et al. 2017). Enhanced schistosomiasis risk may not be limited to areas surrounding the reservoirs but may also extend to upstream catchments hundreds of kilometres away (Sokolow et al. 2017).

The latter examples highlight the positive impact of dam ecosystems on trematode transmission; however, there are also contrasting examples. No significant differences were detected between snails sampled from reservoirs and those from adjacent canals or paddy fields, suggesting that local land use mosaics and connectivity may compensate for reservoir effects on parasite transmission (Le et al. 2025). These findings indicate that reservoirs do not uniformly intensify trematode transmission but rather interact with surrounding habitat types and management practices. Also, after the construction of the Three Gorges Dam in China, the prevalence of schistosomiasis in the middle and lower reaches of the Yangtze River decreased (Zhang et al. 2019).

Beyond trematodes, dams and reservoirs also affect the transmission of vector-borne parasites, notably malaria. Both large and small dams create breeding habitats for mosquitoes by generating shallow, sunlit, and relatively stable water bodies. Evidence indicates that dam-associated increases in malaria transmission are widespread and persistent, particularly in tropical regions (Kibret et al. 2021; Patz et al. 2000). Comparative analyses suggest that small dams may exert even stronger local effects than large dams, with elevated malaria incidence persisting as human population density increases (Kibret et al. 2021). Field studies in northern Ghana demonstrated that communities residing near irrigation dams had significantly higher odds of malaria infection year-round, including the dry seasons when transmission would normally decline. Moreover, individuals living near dams exhibited higher multiplicity of infection, indicating simultaneous infection with multiple *Plasmodium* species, a factor that may complicate disease control and increase the risk of drug resistance (Kyei-Baafour et al. 2020). These findings highlight that dam-induced habitat alteration can stabilize transmission dynamics and undermine seasonal bottlenecks that typically limit parasite spread.

## Land use and agriculture

Agricultural land use represents the most widespread anthropogenic modification of natural ecosystems, profoundly altering habitat conditions by reducing habitat heterogeneity and complexity, decreasing free-living species diversity, and shifting wildlife community composition (Bongaarts 2019; Foley et al. 2005; Schürings et al. 2022). Through cascading ecosystem effects, agricultural land modification can substantially influence infectious disease dynamics and parasite community structure, as exemplified by increased malaria transmission risk following deforestation and rice cultivation (Foley et al. 2005; Lafferty and Kuris 1999; Vittor et al. 2009). Whether parasites benefit from agricultural land use, depends on the type and magnitude of land use modification as well as the life cycle characteristics of the respective parasite. For example, trematode abundance has been shown to increase with expanding crop-field area and associated productivity in adjacent lakes, but to decline once cropland exceeds approximately 50% of the surrounding land use (Hammoud et al. 2025). Agricultural land use and resulting habitat fragmentation may exclude or limit host species required to complete complex parasite life cycles, resulting in reduced parasite diversity or shifts in community composition (Carrera-Jativa and Acosta-Jamett 2023; Hammoud et al. 2025; King et al. 2007; Marcogliese et al. 2009). Consequently, parasite communities in agricultural landscapes are often simplified and dominated by generalist taxa capable of persisting under degraded conditions due to flexible host use (King et al. 2007, 2010; Koprivnikar and Redfern 2012).

Among amphibian-infecting trematodes, agricultural land use has been associated with decreased species richness and shorter life cycles (Gray et al. 2007; King et al. 2010; Marcogliese et al. 2009). However, species-specific increases have been documented for several trematode and nematode species in pasture-dominated landscapes compared to forested areas, indicating greater habitat resources for intermediate hosts involved in the respective life cycles (McKenzie 2007).

Altered host availability and abundance following land use modification are consistent drivers of changes in parasite dynamics within agricultural landscapes. Eutrophication, for example, often leads to an increased abundance of freshwater snail hosts in agricultural wetlands, irrigation channels, and water bodies adjacent to cropland, enhancing transmission of digenean trematodes that depend on snails as first intermediate hosts (Chantima et al. 2018; Grabner et al. 2014; Johnson et al. 2007; Johnson and Carpenter 2010; Mereta et al. 2023). For instance, the abundance of bird schistosomes causing swimmer’s itch in humans increased with eutrophic conditions, driven by higher density, increased growth rates and elevated longevity of first intermediate snail hosts (Soldánová et al. 2013). Fascioliasis in the Nile delta further illustrates amplified parasite transmission through changes in habitat and host availability. In this region, *Fasciola hepatica* and *Fasciola gigantica* are endemic, with *Radix natalensis* serving as the native first intermediate host (Esteban et al. 2003). However, the invasive snail *Pseudosuccinea columella* has also been identified as a suitable host for *F. gigantica* (Grabner et al. 2014). These snails commonly occur in agricultural irrigation channels that are flooded to water crops, facilitating the attachment of *F. gigantica* cercariae to plants and encystment as metacercariae, thereby enhancing transmission to humans and livestock (Grabner et al. 2014). In addition, irrigation channels foster the growth of water hyacinths, which provide habitat for the snails and refuge during molluscicide treatments (Grabner et al. 2014).

The effects of agrochemical exposure on host-parasite interactions are complex and often difficult to predict, with studies reporting contrasting outcomes for parasites and their hosts. Long-term analyses of helminth infections in farmland birds have documented pronounced declines in trematode communities over recent decades, potentially linked to antihelminth activity of widely used agrochemicals (Sitko and Heneberg 2021). Laboratory and field studies further demonstrate that free-living parasitic stages, such as trematode miracidia and cercariae, are particularly sensitive to pesticides and pollutants, resulting in reduced survival or infectivity (Blanar et al. 2009; de Castro Monte et al. 2018; Morley et al. 2003; Pietrock and Marcogliese 2003). In contrast, host-mediated effects of agrochemicals can counteract these impacts, thereby sustaining or even enhancing parasite transmission. For example, glyphosate exposure of the snail *Potamopyrgus antipodarum* has been linked to increased cercarial output for multiple trematode species and enhanced survival for one species (Hock and Poulin 2012), whereas a metabolite of atrazine increased mortality in trematode-infected snails, potentially reducing infection pressure (Koprivnikar and Walker 2011). In a meta-analysis, Hoover et al. (2020) showed that agrochemicals can lead to an increase in human schistosome reproductive ratio and, as a consequence, an increase in disability adjusted life years (DALYs) in the human population due to positive effects for the development of host snail populations or negative effects on snail predators. In amphibians and fish, sublethal pesticide exposure has been shown to reduce immune function and to slow down development, resulting in elevated susceptibility and enhanced infection intensities across helminth and protist parasites (Budischak et al. 2008; Jayawardena et al. 2016, 2017; Kiesecker 2002; Peltzer et al. 2008; Rohr and McCoy 2010; Rohr et al. 2008a, b). For instance, the pesticide atrazine suppresses amphibian immune functions and delays tadpole development. This increases infection rates by plagiorchiid trematodes, ultimately leading to higher mortality rates and amphibian declines (Rohr et al. 2008b; Rumschlag et al. 2019).

## Urbanization

While assessments of urbanization differ depending on definitions and methodological approaches, the global expansion of urban land use is undeniable (e.g., United Nations 2019). Urbanization alters environmental conditions across multiple scales by contributing to climate change, modifying biogeochemical cycles and hydrological regimes, and reshaping ecological communities, often resulting in biodiversity loss (Grimm et al. 2008). These transformations affect not only free-living organisms but also shape host-parasite dynamics, altering host populations as well as parasite transmission (Bradley and Altizer 2007). An example relevant to human health is the urbanization-dependent increase of *Aedes aegypti* abundance due to the increased availability of human hosts and mosquito breeding sites (Montgomery et al. 2025).

In freshwater systems, urban land use has been associated with both reduced and increased parasite abundance, largely depending on host availability and parasite life cycles (e.g., Chapman et al. 2015; Taglioretti et al. 2018; Urban 2006). For example, urban land use has been linked to reduced parasite species richness but increased parasite abundance in pumpkinseed sunfish (*Lepomis gibbosus*) in Ontario streams (Chapman et al. 2015), whereas both fish condition (*Cnesterodon decemmaculatus*) and trematode abundance declined in highly urbanized streams in Argentina (Taglioretti et al. 2018). Parasites featuring disturbance-sensitive hosts or complex life cycles with free-living or ectoparasitic stages may be particularly vulnerable to urbanization, whereas parasites of urban-adapted hosts may benefit from host aggregations and increased transmission success (Altman and Byers 2014; Blanar et al. 2016; Hüsken et al. 2026; Urban 2006). For example, trematodes transmitted by gull definitive hosts were more abundant in trout-perch (*Percopsis omiscomaycus*) at highly urbanized sites, while directly transmitted, pollution-sensitive monogeneans declined with increasing contaminant loads in urban habitats (Blanar et al. 2016), even though the same stressors were acting in both cases. Similarly, heteroxenous parasites of the killifish *Fundulus heteroclitus* declined significantly with increasing degree of urbanization (Alfieri and Anderson 2019).

Chemical pollution represents a major pathway by which urbanization influences parasite dynamics. Urban runoff introduces contaminants such as metals, pesticides, and polycyclic aromatic hydrocarbons (PAHs) into aquatic systems (Cojoc et al. 2024), which can exert pronounced negative effects on directly exposed ectoparasites or free-living transmission stages (reviewed in Leiva et al. 2024; Sures et al. 2017b). In addition, road de-icing and wastewater input contribute to freshwater salinization (Le et al. 2018). Yet, parasite-host dynamics respond inconsistently to anthropogenic salinization. Elevated salinity has been reported to both decrease and increase survival and transmission of trematode cercariae (Donnelly et al. 1984; Milotic et al. 2017, 2020; Prasopdee et al. 2020; Yu et al. 2022), but salt might increase host susceptibility under saline stress, thus increasing net transmission, instead of directly impairing cercarial transmission (Milotic et al. 2017, 2020). Another important urban stressor that is increasingly recognized is artificial light at night along coastlines and in riverine systems (Liu et al. 2024; Marangoni et al. 2022). Disruption of natural diel rhythms may substantially alter host activity, immune function, timing of parasite transmission, and phototaxis of infectious stages (reviewed in Poulin 2023). Pervasive light pollution can thus enhance parasite transmission and infection, for example, through aggregation of hosts and parasites or increased trophic interactions (MacAulay and Cable 2024; Poulin 2023; Tüzün et al. 2025), but may also suppress transmission in nocturnal vectors or negatively phototactic stages, as demonstrated for *Plasmodium* spp. in malaria vectors (e.g., Coetzee and van Zyl 2025; Llergo et al. 2024).

The increase in impervious surfaces in urban habitats promotes surface runoff, whereas channelization, water abstraction, and climate-driven changes in precipitation additionally alter flow regimes of urban streams (Debbage and Shepherd 2018). Reduced flow conditions are typically associated with enhanced parasite prevalence and transmission due to increasing encounter rates between hosts and infectious stages (reviewed in Marcogliese 2001). However, higher runoff associated with increased precipitation has been linked to higher parasite diversity but lower abundance in spottail shiners (*Notropis hudsonius*) (Marcogliese et al. 2016). Furthermore, the expansion of sealed surface areas can increase urban flood risks and may contribute to increased infection levels, as illustrated by schistosomiasis outbreaks in the human population in Brazil (Barbosa et al. 2011; De Oliveira et al. 2020).

## Conclusion

Human-induced habitat alteration has profoundly reshaped freshwater ecosystems, with cascading effects on host-parasite dynamics that can extend far beyond individual species or localities. As demonstrated by the specific cases addressed in this review, from the restoration of the Emscher River to the ecological consequences of dams, agriculture, and urbanization, parasites respond in different ways to habitat alterations. While some parasites thrive under altered conditions, benefiting from host aggregation, eutrophication, or vector proliferation, others decline due to disrupted life cycles, habitat fragmentation, or chemical stressors. These shifts can carry significant implications for biodiversity, public health, and ecosystem functioning. For instance, the rise of trematode-borne diseases in reservoir systems or the amplification of malaria near irrigation dams underscore how infrastructure and land use decisions can inadvertently exacerbate disease risks. Conversely, the loss of parasite diversity, particularly among taxa with complex life cycles, may reflect broader declines in species diversity and ecosystem resilience, as parasites play critical roles in food web stability, nutrient cycling, and population regulation.

In many cases, habitat alteration simplifies parasite communities, favouring generalists and disturbance-tolerant taxa, while it can simultaneously create novel transmission pathways for some parasites that elevate infection risks for humans, livestock, and wildlife. This duality highlights the need for integrated approaches to ecosystem management that consider parasites not only as pathogens but as integral components of ecological networks. Restoration efforts, such as those in the Emscher catchment, offer promising examples of how targeted interventions can revive parasite diversity and host-parasite interactions, though persistent stressors may limit full recovery. Similarly, the design of dams, agricultural practices, and urban infrastructure must account for their unintended consequences on parasite transmission, balancing human needs with ecological sustainability.

Moving forward, a One Health perspective that bridges ecology, parasitology, and public health will be essential for anticipating and mitigating the impacts of habitat alteration on host-parasite systems. As global change accelerates, understanding the responses of parasites to environmental transformation will not only deepen our knowledge of ecosystem functioning but also help protect both biodiversity and human well-being in an increasingly human-dominated world.

## Data Availability

No datasets were generated or analysed during the current study.

## References

[CR1] Alfieri JM, Anderson TK (2019) Life-cycle mediated effects of urbanization on parasite communities in the estuarine fish, Fundulus heteroclitus. PLoS One 14(12):e0225896. 10.1371/journal.pone.0225896 31790480 10.1371/journal.pone.0225896 PMC6886805

[CR2] Altman I, Byers JE (2014) Large-scale spatial variation in parasite communities influenced by anthropogenic factors. Ecology 95(7):1876–87. 10.1890/13-0509.1 25163120 10.1890/13-0509.1

[CR3] Barbosa CS, Leal-Neto OB, Gomes EC, Araujo KC, Domingues AL (2011) The endemisation of schistosomiasis in Porto de Galinhas, Pernambuco, Brazil, 10 years after the first epidemic outbreak. Mem Inst Oswaldo Cruz 106(7):878–883. 10.1590/s0074-02762011000700014 22124561 10.1590/s0074-02762011000700014

[CR4] Blanar CA, Munkittrick KR, Houlahan J, MacLatchy DL, Marcogliese DJ (2009) Pollution and parasitism in aquatic animals: A meta-analysis of effect size. Aquat Toxicol 93(1):18–28. 10.1016/j.aquatox.2009.03.002 19349083 10.1016/j.aquatox.2009.03.002

[CR5] Blanar CA et al (2016) Parasite community similarity in Athabasca River trout-perch (*Percopsis omiscomaycus*) varies with local-scale land use and sediment hydrocarbons, but not distance or linear gradients. Parasitol Res 115(10):3853–3866. 10.1007/s00436-016-5151-x 27314231 10.1007/s00436-016-5151-x

[CR6] Bongaarts J (2019) Summary for policymakers of the global assessment report on biodiversity and ecosystem services of the Intergovernmental Science-Policy Platform on Biodiversity and Ecosystem Services. Popul Dev Rev 45(3):680–681. 10.1111/padr.12283

[CR7] Bradley CA, Altizer S (2007) Urbanization and the ecology of wildlife diseases. Trends Ecol Evol 22(2):95–102. 10.1016/j.tree.2006.11.001 17113678 10.1016/j.tree.2006.11.001 PMC7114918

[CR8] Budischak SA, Belden LK, Hopkins WA (2008) Effects of malathion on embryonic development and latent susceptibility to trematode parasites in ranid tadpoles. Environ Toxicol Chem 27(12):2496–2500. 10.1897/08-018.1 18613741 10.1897/08-018.1

[CR9] Cable J et al (2017) Global change, parasite transmission and disease control: lessons from ecology. Philos Trans R Soc Lond B Biol Sci. 10.1098/rstb.2016.0088 28289256 10.1098/rstb.2016.0088 PMC5352815

[CR10] Carrera-Jativa PD, Acosta-Jamett G (2023) Influence of habitat alteration on the structure of helminth communities in small mammals: a systematic review and critical appraisal of theory and current evidence. Parasitol Res 122(5):1053–1070. 10.1007/s00436-023-07804-8 36894783 10.1007/s00436-023-07804-8

[CR11] Chantima K, Suk-Ueng K, Kampan M (2018) Freshwater snail diversity in Mae Lao agricultural basin (Chiang Rai, Thailand) with a focus on larval trematode infections. Korean J Parasitol 56(3):247–257. 10.3347/kjp.2018.56.3.247 29996628 10.3347/kjp.2018.56.3.247 PMC6046552

[CR12] Chapman JM, Marcogliese DJ, Suski CD, Cooke SJ (2015) Variation in parasite communities and health indices of juvenile *Lepomis gibbosus* across a gradient of watershed land-use and habitat quality. Ecol Indic 57:564–572. 10.1016/j.ecolind.2015.05.013

[CR13] Coetzee BWT, van Zyl L (2025) How much does light pollution alter vector disease transmission at scale? Afr J Ecol. 10.1111/aje.70067

[CR14] Cojoc L et al (2024) Pollutants in urban runoff: scientific evidence on toxicity and impacts on freshwater ecosystems. Chemosphere 369:143806. 10.1016/j.chemosphere.2024.143806 39603359 10.1016/j.chemosphere.2024.143806

[CR15] de Castro Monte TCC et al (2018) Morphological effects on helminth parasites caused by herbicide under experimental conditions. Brazilian J Veterinary Parasitol 27(1):42–51. 10.1590/S1984-29612017074 10.1590/S1984-29612017074 29641790

[CR16] De Oliveira ECA, Da Silva IEP, Ferreira RJ, Guimaraes R, Gomes ECS, Barbosa CS (2020) Mapping the risk for transmission of urban schistosomiasis in the Brazilian Northeast. Geospat Health. 10.4081/gh.2020.861 33461283 10.4081/gh.2020.861

[CR17] Debbage N, Shepherd JM (2018) The influence of urban development patterns on streamflow characteristics in the Charlanta megaregion. Water Resour Res 54(5):3728–3747. 10.1029/2017wr021594

[CR18] Diakité NR, Winkler MS, Coulibaly JT, Guindo-Coulibaly N, Utzinger J, N’Goran EK (2017) Dynamics of freshwater snails and Schistosoma infection prevalence in schoolchildren during the construction and operation of a multipurpose dam in central Cote d’Ivoire. Infect Dis Poverty 6(1):93. 10.1186/s40249-017-0305-3 28468667 10.1186/s40249-017-0305-3 PMC5415719

[CR19] Donnelly FA, Appleton CC, Schutte CH (1984) The influence of salinity on the cercariae of three species of *Schistosoma*. Int J Parasitol 14(1):13–21. 10.1016/0020-7519(84)90005-5 6706462 10.1016/0020-7519(84)90005-5

[CR20] Ellis EC (2021) Land Use and Ecological Change: A 12,000-Year History. Annu Rev Environ Resour 46(1):1–33. 10.1146/annurev-environ-012220-010822

[CR21] Esch GW, Kennedy CR, Bush AO, Aho JM (1988) Patterns in helminth communities in freshwater fish in Great Britain: alternative strategies for colonization. Parasitology 96(Pt 3):519–532. 10.1017/s003118200008015x 3405638 10.1017/s003118200008015x

[CR22] Esteban JG et al (2003) Hyperendemic fascioliasis associated with schistosomiasis in villages in the Nile Delta of Egypt. Am J Trop Med Hyg 69(4):429–437 14640504

[CR23] Foley JA et al (2005) Global consequences of land use. Science 309(5734):570–574. 10.1126/science.1111772 16040698 10.1126/science.1111772

[CR24] Gillmann SM, Hering D, Lorenz AW (2023) Habitat development and species arrival drive succession of the benthic invertebrate community in restored urban streams. Environ Sci Eur 35:49. 10.1186/s12302-023-00756-x

[CR25] Gomez A, Nichols E (2013) Neglected wild life: Parasitic biodiversity as a conservation target. Int J Parasitol Parasites Wildl 2:222–227. 10.1016/j.ijppaw.2013.07.002 24533340 10.1016/j.ijppaw.2013.07.002 PMC3862516

[CR26] Gottdenker NL, Streicker DG, Faust CL, Carroll CR (2014) Anthropogenic land use change and infectious diseases: a review of the evidence. EcoHealth 11(4):619–632. 10.1007/s10393-014-0941-z 24854248 10.1007/s10393-014-0941-z

[CR27] Grabner DS, Mohamed FA, Nachev M, Meabed EM, Sabry AH, Sures B (2014) Invasion biology meets parasitology: a case study of parasite spill-back with Egyptian *Fasciola gigantica* in the invasive snail *Pseudosuccinea columella*. PLoS One 9(2):e88537. 10.1371/journal.pone.0088537 24523913 10.1371/journal.pone.0088537 PMC3921205

[CR28] Gray MJ, Smith LM, Miller DL, Bursey CR (2007) Influences of agricultural land use on *Clinostomum attenuatum* metacercariae prevalence in Southern Great Plains amphibians, USA. Herpetol Conserv Bio 2(1):23–28

[CR29] Grimm NB et al (2008) Global change and the ecology of cities. Science 319(5864):756–760. 10.1126/science.1150195 18258902 10.1126/science.1150195

[CR30] Haase P et al (2023) The recovery of European freshwater biodiversity has come to a halt. Nature 620(7974):582–588. 10.1038/s41586-023-06400-1 37558875 10.1038/s41586-023-06400-1 PMC10432276

[CR31] Hammoud C et al (2025) Agricultural land use and ensuing eutrophication both shape parasitic trematode communities in rural African lakes. Proc Biol Sci 292(2048):20250070. 10.1098/rspb.2025.0070 40461070 10.1098/rspb.2025.0070 PMC12133380

[CR32] Hock SD, Poulin R (2012) Exposure of the snail *Potamopyrgus antipodarum* to herbicide boosts output and survival of parasite infective stages. Int J Parasitol Parasites Wildl 1:13–18. 10.1016/j.ijppaw.2012.10.002 24533309 10.1016/j.ijppaw.2012.10.002 PMC3904088

[CR33] Hoover CM et al (2020) Effects of agrochemical pollution on schistosomiasis transmission: a systematic review and modelling analysis. Lancet Planet Health 4(7):e280–e291. 10.1016/S2542-5196(20)30105-4 32681899 10.1016/S2542-5196(20)30105-4 PMC7754781

[CR34] Hüsken A, Schwelm J, Sures B (2026) Land use drives trematode dynamics in a restored stream system. Curr Res Parasitol Vector Borne Dis 9:100357. 10.1016/j.crpvbd.2026.100357 41783589 10.1016/j.crpvbd.2026.100357 PMC12954508

[CR35] Jayawardena UA, Rohr JR, Navaratne AN, Amerasinghe PH, Rajakaruna RS (2016) Combined effects of pesticides and trematode infections on hourglass tree frog *Polypedates cruciger*. EcoHealth 13(1):111–122. 10.1007/s10393-016-1103-2 26911919 10.1007/s10393-016-1103-2 PMC4852980

[CR36] Jayawardena UA, Rohr JR, Amerasinghe PH, Navaratne AN, Rajakaruna RS (2017) Effects of agrochemicals on disease severity of *Acanthostomum burminis* infections (Digenea: Trematoda) in the Asian common toad, *Duttaphrynus melanostictus*. BMC Zool. 10.1186/s40850-017-0022-1

[CR37] Johnson PTJ, Carpenter SR (2010) Influence of eutrophication on disease in aquatic ecosystems: Patterns, processes, and predictions. In: Ostfeld RS, Keesing F, Eviner VT (eds) Infectious disease ecology: the effects of ecosystems on disease and of disease on ecosystems. Princeton University Press, p p 71-99

[CR38] Johnson PT et al (2007) Aquatic eutrophication promotes pathogenic infection in amphibians. Proc Natl Acad Sci U S A 104(40):15781–15786. 10.1073/pnas.0707763104 17893332 10.1073/pnas.0707763104 PMC2000446

[CR39] Kibret S, McCartney M, Lautze J, Nhamo L, Yan G (2021) The impact of large and small dams on malaria transmission in four basins in Africa. Sci Rep 11(1):13355. 10.1038/s41598-021-92924-3 34172779 10.1038/s41598-021-92924-3 PMC8233325

[CR40] Kiesecker JM (2002) Synergism between trematode infection and pesticide exposure: a link to amphibian limb deformities in nature? Proc Natl Acad Sci U S A 99(15):9900–9904. 10.1073/pnas.152098899 12118118 10.1073/pnas.152098899 PMC126596

[CR41] King KC et al (2007) Impacts of agriculture on the parasite communities of northern leopard frogs (*Rana pipiens*) in southern Quebec, Canada. Parasitology 134(Pt.14):2063–80. 10.1017/S0031182007003277 17672926 10.1017/S0031182007003277

[CR42] King KC, Daniel Mclaughlin J, Boily M, Marcogliese DJ (2010) Effects of agricultural landscape and pesticides on parasitism in native bullfrogs. Biol Conserv 143(2):302–310. 10.1016/j.biocon.2009.10.011

[CR43] Koprivnikar J, Redfern JC (2012) Agricultural effects on amphibian parasitism: importance of general habitat perturbations and parasite life cycles. J Wildl Dis 48(4):925–36. 10.7589/2011-09-258 23060494 10.7589/2011-09-258

[CR44] Koprivnikar J, Walker PA (2011) Effects of the herbicide atrazine’s metabolites on host snail mortality and production of trematode cercariae. J Parasitol 97(5):822–7. 10.1645/GE-2814.1 21554070 10.1645/GE-2814.1

[CR45] Kyei-Baafour E et al (2020) Impact of an irrigation dam on the transmission and diversity of *Plasmodium falciparum* in a seasonal malaria transmission area of Northern Ghana. J Trop Med 2020:1386587. 10.1155/2020/1386587 32308690 10.1155/2020/1386587 PMC7155757

[CR46] Lafferty KD, Kuris AM (1999) How environmental stress affects the impacts of parasites. Limnol Oceanogr 44(3):925–931

[CR48] Le AH et al (2025) Hydropower reservoirs - their potential association with transmission of trematodes in Vietnam. J Helminthol 99:e23. 10.1017/S0022149X2400097X 39924657 10.1017/S0022149X2400097X

[CR47] Le TDH, Kattwinkel M, Schutzenmeister K, Olson JR, Hawkins CP, Schäfer RB (2018) Predicting current and future background ion concentrations in German surface water under climate change. Philos Trans R Soc Lond B Biol Sci. 10.1098/rstb.2018.0004 30509906 10.1098/rstb.2018.0004 PMC6283972

[CR49] Leiva NV, Montenegro D, Orrego R, Vidal R, Gonzalez MT (2024) Tolerance of free-living larval stage of a parasite from coastal mining areas in northern Humboldt current to copper pollution at low and high temperatures. PLoS One 19(11):e0310473. 10.1371/journal.pone.0310473 39499694 10.1371/journal.pone.0310473 PMC11537404

[CR50] Liu Y, Huang Y, Liu Y, Liu S, Yao L, Cao D (2024) Do rivers get sufficient sleep—a global analysis of light pollution in rivers. Resour Conserv Recycl. 10.1016/j.resconrec.2024.107892

[CR51] Llergo JL, Garuti H, Lopez C, Sanchez J, Calvo D (2024) Artificial nighttime lighting impacts *Plasmodium falciparum* mature stage V gametocytes infectivity in *Anopheles stephensi*. Malar J 23(1):42. 10.1186/s12936-024-04866-6 38326842 10.1186/s12936-024-04866-6 PMC10851600

[CR52] Louvard C, Hadfield KA, Vanhove MPM, Sures B, Smit NJ (2025) Unveiling the Hidden Players: Exploring the Intricate Dance of Aquatic Parasites, Host Biodiversity and Ecosystem Health. In: Smit NJ, Sures B (eds) Aquatic Parasitology: Ecological and Environmental Concepts and Implications of Marine and Freshwater Parasites. Springer, Cham, pp 167–198

[CR53] MacAulay S, Cable J (2024) *Gyrodactylus* in the spotlight: how exposure to light impacts disease and the feeding behavior of the freshwater tropical guppy (*Poecilia reticulata*). J Fish Biol 105(3):682–690. 10.1111/jfb.15816 38828698 10.1111/jfb.15816

[CR54] Marangoni LFB et al (2022) Impacts of artificial light at night in marine ecosystems-A review. Glob Chang Biol 28(18):5346–5367. 10.1111/gcb.16264 35583661 10.1111/gcb.16264 PMC9540822

[CR55] Marcogliese DJ (2001) Implications of climate change for parasitism of animals in the aquatic environment. Can J Zool 79(8):1331–1352. 10.1139/z01-067

[CR56] Marcogliese DJ (2004) Parasites: Small Players with Crucial Roles in the Ecological Theater. EcoHealth 1(2):151–164. 10.1007/s10393-004-0028-3

[CR57] Marcogliese DJ (2005) Parasites of the superorganism: are they indicators of ecosystem health? Int J Parasitol 35(7):705–716. 10.1016/j.ijpara.2005.01.015 15925594 10.1016/j.ijpara.2005.01.015

[CR58] Marcogliese DJ et al (2009) Combined effects of agricultural activity and parasites on biomarkers in the bullfrog, *Rana catasbeiana*. Aquat Toxicol 91(2):126–134. 10.1016/j.aquatox.2008.10.001 19019467 10.1016/j.aquatox.2008.10.001

[CR59] Marcogliese DJ, Locke SA, Gelinas M, Gendron AD (2016) Variation in parasite communities in spottail shiners (*Notropis hudsonius*) linked with precipitation. J Parasitol 102(1):27–36. 10.1645/12-31 26465386 10.1645/12-31

[CR60] McKenzie VJ (2007) Human land use and patterns of parasitism in tropical amphibian hosts. Biol Conserv 137(1):102–116. 10.1016/j.biocon.2007.01.019

[CR61] Mereta ST et al (2023) Effects of land-use and environmental factors on snail distribution and trematode infection in Ethiopia. Trop Med Infect Dis. 10.3390/tropicalmed8030154 36977155 10.3390/tropicalmed8030154 PMC10053549

[CR62] Milotic D, Milotic M, Koprivnikar J (2017) Effects of road salt on larval amphibian susceptibility to parasitism through behavior and immunocompetence. Aquat Toxicol 189:42–49. 10.1016/j.aquatox.2017.05.015 28582700 10.1016/j.aquatox.2017.05.015

[CR63] Milotic D, Milotic M, Koprivnikar J (2020) Effects of road salt on a free-living trematode infectious stage. J Helminthol 94:e150. 10.1017/S0022149X20000309 32381146 10.1017/S0022149X20000309

[CR64] Montgomery MJ et al (2025) The effects of urbanization, temperature, and rainfall on *Aedes aegypti* and *Aedes albopictus* mosquito abundance across a broad latitudinal gradient in Central Africa. Parasit Vectors 18(1):135. 10.1186/s13071-025-06764-5 40189559 10.1186/s13071-025-06764-5 PMC11972486

[CR65] Morley NJ, Irwin SW, Lewis JW (2003) Pollution toxicity to the transmission of larval digeneans through their molluscan hosts. Parasitol 126 Suppl S5–26. 10.1017/s0031182003003755 10.1017/s0031182003003755 14667169

[CR66] Ong X, Wang Y-C, Sithithaworn P, Grundy-Warr C, Pitaksakulrat O (2016) Dam influences on liver fluke transmission: Fish infection and human fish consumption behavior. Annals Am Association Geographers 106(4):755–772. 10.1080/24694452.2015.1122508

[CR67] Patz JA, Graczyk TK, Geller N, Vittor AY (2000) Effects of environmental change on emerging parasitic diseases. Int J Parasitol 30(12–13):1395–1405. 10.1016/s0020-7519(00)00141-7 11113264 10.1016/s0020-7519(00)00141-7

[CR68] Peltzer PM, Lajmanovich RC, Sanchez-Hernandez JC, Cabagna MC, Attademo AM, Basso A (2008) Effects of agricultural pond eutrophication on survival and health status of *Scinax nasicus* tadpoles. Ecotoxicol Environ Saf 70(1):185–97. 10.1016/j.ecoenv.2007.06.005 17658602 10.1016/j.ecoenv.2007.06.005

[CR69] Pietrock M, Marcogliese DJ (2003) Free-living endohelminth stages: at the mercy of environmental conditions. Trends Parasitol 19(7):293–299. 10.1016/s1471-4922(03)00117-x 12855379 10.1016/s1471-4922(03)00117-x

[CR70] Poulin R (2023) Light pollution may alter host-parasite interactions in aquatic ecosystems. Trends Parasitol 39(12):1050–1059. 10.1016/j.pt.2023.08.013 37722935 10.1016/j.pt.2023.08.013

[CR71] Prasopdee S, Kulsantiwong J, Sathavornmanee T, Thitapakorn V (2020) The effects of temperature and salinity on the longevity of *Opisthorchis viverrini* cercariae: a climate change concern. J Helminthol 94:e165. 10.1017/S0022149X20000498 32571436 10.1017/S0022149X20000498

[CR72] Prati S, Grabner DS, Pfeifer SM, Lorenz AW, Sures B (2022) Generalist parasites persist in degraded environments: a lesson learned from microsporidian diversity in amphipods. Parasitology 149(7):1–10. 10.1017/S0031182022000452 10.1017/S0031182022000452 PMC10090640 35485747

[CR73] Rohr JR, McCoy KA (2010) A qualitative meta-analysis reveals consistent effects of atrazine on freshwater fish and amphibians. Environ Health Perspect 118(1):20–32. 10.1289/ehp.0901164 20056568 10.1289/ehp.0901164 PMC2831963

[CR74] Rohr JR, Raffel TR, Sessions SK, Hudson PJ (2008a) Understanding the net effects of pesticides on amphibian trematode infections. Ecol Appl 18(7):1743–53. 10.1890/07-1429.1 18839768 10.1890/07-1429.1

[CR75] Rohr JR et al (2008b) Agrochemicals increase trematode infections in a declining amphibian species. Nature 455(7217):1235–1239. 10.1038/nature07281 18972018 10.1038/nature07281

[CR76] Rumschlag SL et al (2019) Effects of pesticides on exposure and susceptibility to parasites can be generalised to pesticide class and type in aquatic communities. Ecol Lett 22(6):962–972. 10.1111/ele.13253 30895712 10.1111/ele.13253 PMC6483824

[CR77] Schürings C, Feld CK, Kail J, Hering D (2022) Effects of agricultural land use on river biota: a meta-analysis. Environ Sci Eur 34(1):124. 10.1186/s12302-022-00706-z

[CR78] Schürings C et al (2025) Drivers of recovery and degradation of riverine benthic invertebrate communities: a Germany-wide analysis. Ecol Processes 14(1):30. 10.1186/s13717-025-00593-1

[CR79] Seidel M, Li F, Winking C, Sommerhäuser M, Lüderitz V (2022) Should we sample more than required by the European Water Framework Directive? Case study: Emscher catchment. Clean Soil Air Water. 10.1002/clen.202000391

[CR80] Selbach C, Soldánová M, Feld CK, Kostadinova A, Sures B (2020) Hidden parasite diversity in a European freshwater system. Sci Rep 10(1):2694. 10.1038/s41598-020-59548-5 32060320 10.1038/s41598-020-59548-5 PMC7021786

[CR81] Sitko J, Heneberg P (2021) Long-term dynamics of trematode infections in common birds that use farmlands as their feeding habitats. Parasit Vectors 14(1):383. 10.1186/s13071-021-04876-2 34353362 10.1186/s13071-021-04876-2 PMC8344216

[CR82] Sokolow SH et al (2017) Nearly 400 million people are at higher risk of schistosomiasis because dams block the migration of snail-eating river prawns. Philos Trans R Soc Lond B Biol Sci. 10.1098/rstb.2016.0127 28438916 10.1098/rstb.2016.0127 PMC5413875

[CR84] Soldánová M, Selbach C, Kalbe M, Kostadinova A, Sures B (2013) Swimmer’s itch: etiology, impact, and risk factors in Europe. Trends Parasitol 29(2):65–74. 10.1016/j.pt.2012.12.002 23305618 10.1016/j.pt.2012.12.002

[CR83] Soldánová M, Selbach C, Sures B, Kostadinova A, Perez-Del-Olmo A (2010) Larval trematode communities in *Radix auricularia* and *Lymnaea stagnalis* in a reservoir system of the Ruhr River. Parasites Vectors 3:56. 10.1186/1756-3305-3-56 20576146 10.1186/1756-3305-3-56 PMC2910012

[CR85] Sures B, Nachev M, Pahl M, Grabner D, Selbach C (2017a) Parasites as drivers of key processes in aquatic ecosystems: Facts and future directions. Exp Parasitol 180:141–147. 10.1016/j.exppara.2017.03.011 28456692 10.1016/j.exppara.2017.03.011

[CR86] Sures B, Nachev M, Selbach C, Marcogliese DJ (2017b) Parasite responses to pollution: what we know and where we go in “Environmental Parasitology.” Parasit Vectors 10(1):65. 10.1186/s13071-017-2001-3 28166838 10.1186/s13071-017-2001-3 PMC5294906

[CR87] Sures B, Nachev M, Schwelm J, Grabner D, Selbach C (2023) Environmental parasitology: stressor effects on aquatic parasites. Trends Parasitol 39(6):461–474. 10.1016/j.pt.2023.03.005 37061443 10.1016/j.pt.2023.03.005

[CR88] Taglioretti V, Rossin MA, Timi JT (2018) Fish-trematode systems as indicators of anthropogenic disturbance: Effects of urbanization on a small stream. Ecol Indic 93:759–770. 10.1016/J.ECOLIND.2018.05.039

[CR89] Titcomb G, Mantas JN, Hulke J, Rodriguez I, Branch D, Young H (2021) Water sources aggregate parasites with increasing effects in more arid conditions. Nat Commun 12(1):7066. 10.1038/s41467-021-27352-y 34862389 10.1038/s41467-021-27352-y PMC8642388

[CR90] Tüzün N, Holker F, De Meester L (2025) Artificial light at night intensifies effects of a parasitic flatworm on the water flea *Daphnia magna*. Biol Lett 21(9):20250373. 10.1098/rsbl.2025.0373 40987336 10.1098/rsbl.2025.0373 PMC12457030

[CR91] United Nations, Department of Economic and Social Affairs, Population Division (2019) World Urbanization Prospects: The 2018 Revision, ST/ESA/SER.A/420. United Nations, New York

[CR92] Urban MC (2006) Road facilitation of trematode infections in snails of northern Alaska. Conserv Biol 20(4):1143–1149. 10.1111/j.1523-1739.2006.00422.x 16922230 10.1111/j.1523-1739.2006.00422.x

[CR93] Vittor AY et al (2009) Linking deforestation to malaria in the Amazon: characterization of the breeding habitat of the principal malaria vector, *Anopheles darlingi*. Am J Trop Med Hyg 81(1):5–12 19556558 PMC3757555

[CR94] Vos M et al (2023) The asymmetric response concept explains ecological consequences of multiple stressor exposure and release. Sci Total Environ 872:162196. 10.1016/j.scitotenv.2023.162196 36781140 10.1016/j.scitotenv.2023.162196

[CR95] Winking C, Lorenz AW, Sures B, Hering D (2014) Recolonisation patterns of benthic invertebrates: a field investigation of restored former sewage channels. Freshwat Biol 59(9):1932–1944. 10.1111/fwb.12397

[CR96] Wood CL (2024) Parasites in a changing world: Troublesome or in trouble? Annu Rev Anim Biosci 13:303–323. 10.1146/annurev-animal-111523-102039 39527716 10.1146/annurev-animal-111523-102039

[CR97] Wood CL et al (2023) A reconstruction of parasite burden reveals one century of climate-associated parasite decline. Proc Natl Acad Sci U S A 120(3):e2211903120. 10.1073/pnas.2211903120 36623180 10.1073/pnas.2211903120 PMC9934024

[CR98] Yu A, Vannatta JT, Gutierrez SO, Minchella DJ (2022) Opportunity or catastrophe? Effect of sea salt on host-parasite survival and reproduction. PLoS Negl Trop Dis 16(2):e0009524. 10.1371/journal.pntd.0009524 35202408 10.1371/journal.pntd.0009524 PMC8870500

[CR99] Zhang S et al (2019) Influence of the Three Gorges Dam on schistosomiasis control in the middle and lower reaches of the Yangtze River. Global Health J 3(1):9–15. 10.1016/j.glohj.2019.03.003

[CR100] Ziegler AD et al (2013) Dams and disease triggers on the lower Mekong river. PLoS Negl Trop Dis 7(6):e2166. 10.1371/journal.pntd.0002166 23853695 10.1371/journal.pntd.0002166 PMC3682813

